# Compensating Positron Range Effects of Ga-68 in Preclinical PET Imaging by Using Convolutional Neural Network: A Monte Carlo Simulation Study

**DOI:** 10.3390/diagnostics11122275

**Published:** 2021-12-04

**Authors:** Ching-Ching Yang

**Affiliations:** 1 Department of Medical Imaging and Radiological Sciences, Kaohsiung Medical University, Kaohsiung 807, Taiwan; cyang@kmu.edu.tw; 2 Department of Medical Research, Kaohsiung Medical University Chung-Ho Memorial Hospital, Kaohsiung 807, Taiwan

**Keywords:** Ga-68 preclinical PET imaging, positron range correction, convolutional neural network

## Abstract

This study aimed to investigate the feasibility of positron range correction based on three different convolutional neural network (CNN) models in preclinical PET imaging of Ga-68. The first model (CNN1) was originally designed for super-resolution recovery, while the second model (CNN2) and the third model (CNN3) were originally designed for pseudo CT synthesis from MRI. A preclinical PET scanner and 30 phantom configurations were modeled in Monte Carlo simulations, where each phantom configuration was simulated twice, once for Ga-68 (CNN input images) and once for back-to-back 511-keV gamma rays (CNN output images) with a 20 min emission scan duration. The Euclidean distance was used as the loss function to minimize the difference between CNN input and output images. According to our results, CNN3 outperformed CNN1 and CNN2 qualitatively and quantitatively. With regard to qualitative observation, it was found that boundaries in Ga-68 images became sharper after correction. As for quantitative analysis, the recovery coefficient (RC) and spill-over ratio (SOR) were increased after correction, while no substantial increase in coefficient of variation of RC (CV_RC_) or coefficient of variation of SOR (CV_SOR_) was observed. Overall, CNN3 should be a good candidate architecture for positron range correction in Ga-68 preclinical PET imaging.

## 1. Introduction

Positron emission tomography (PET) is widely recognized as a powerful imaging technique for in vivo quantification and localization of physiological and pathophysiological functions. Furthermore, PET imaging allows to follow the progression of human diseases in transgenic and knockout mice noninvasively, so it has been used to study the effectiveness of new drugs or treatments [1,2,3]. Due to the small size of experimental animals, high spatial resolution is mandatory in preclinical PET system, which is associated with positron physics, scanner design, data correction, and the reconstruction algorithm [4,5]. Among various positron emission radioisotopes, F-18 is by far the most widely used radionuclide. Nevertheless, with the increasing interest in theranostic approaches for cancer treatment, radioisotopes other than F-18 are also considered in PET imaging, such as Ga-68 [6,7,8]. Using Ga-68 labeled tracers for diagnostics can be effectively followed by targeted radionuclide therapy performed using the same tracer labeled with Lu-177. Since Ga-68 PET imaging is used to determine the therapeutic protocols with Lu-177, the dose delivered to targets and organs at risk through Lu-177 radionuclide therapy is affected by the imaging performance of Ga-68 PET [9,10,11]. The mean positron energy of Ga-68 is 0.83 MeV, which results in a mean positron range of 3.5 mm. Hence, the spatial resolution of PET imaging is inferior with Ga-68 compared to F-18 [12]. Improving spatial resolution through positron range correction would increase the accuracy of Ga-68 PET-based treatment planning. Convolutional neural network (CNN) has been applied in several medical imaging areas, and various architectures have been developed for different tasks [13]. This study aimed to investigate the feasibility of positron range correction based on three different CNN models in preclinical PET imaging of Ga-68. The first CNN model (CNN1) used in this study was originally designed to recover high resolution image from low resolution input image, while the second CNN model (CNN2) and the third CNN model (CNN3) were originally designed to convert MRI into pseudo CT. The image data for CNN training and testing were generated by Monte Carlo simulation to prevent experimental errors while realistically modeling the positron range effects.

## 2. Materials and Methods

### 2.1. Monte Carlo Simulation Toolkit

Monte Carlo simulation was performed by using GATE (GEANT4 Application for Tomographic Emission) version 6.0.0 [14]. GATE comprises four layers of codes, which is GEANT4 in the innermost layer, followed by the core layer, application layer, and user layer. GEANT4, a toolkit for the simulation of the passage of particles through matter based on Monte Carlo method, has been proven to be a proper tool for simulation of positron transportation. In GATE, the scanner geometry, particle type, position, energy, physical interactions of particles with matter and run process were defined by using a scripted language at the user layer to output descriptive data in the form of random number for running simulation using GEANT4.

### 2.2. Preclinical PET System

A FLEX Triumph PET/CT scanner (Gamma Medica-Ideas, Nortridge, CA, USA) was modeled by using the cylindrical PET system in GATE, which was comprised of 5 hierarchic levels: world cylindrical PET, r sector, module, crystal, layer, to produce and store the hit information that generates the singles and the coincidences of the simulation. The preclinical PET scanner investigated in this study includes 180 detector blocks that are arranged into 48 rings, and each block contains an 8 × 8 array of BGO crystals of 2.3 × 2.3 × 10 mm^3^. This configuration covers a transaxial field-of-view (FOV) of 10 cm and an axial FOV of 11.6 cm. PET data were simulated with a 250- to 750-keV energy window and 12-ns timing window in listmode format, which were consequently assigned into 3D sinograms. The sinograms were Fourier rebinned first and then reconstructed using 2D ordered subsets expectation maximization with 4 iterations and 10 subsets. The voxel size used for PET reconstruction was 0.4 × 0.4 × 0.4 mm^3^.

### 2.3. Phantom Design

**Figure 1:**
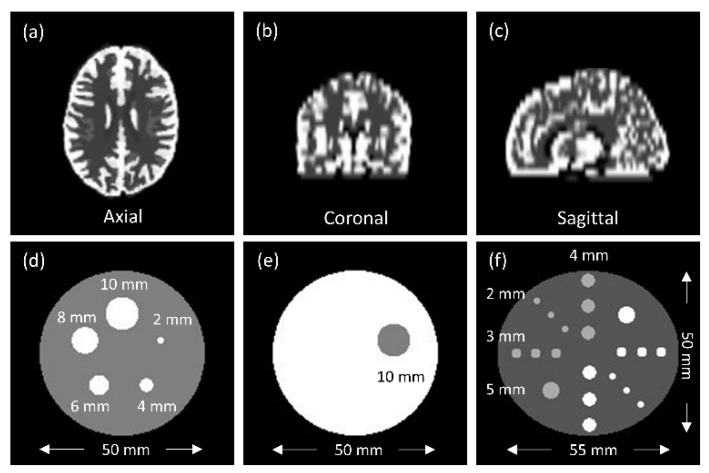
PHAN_brain_ in (**a**) axial plane, (**b**) coronal plane, (**c**) sagittal plane, and the central axial slice of (**d**) PHAN_5rod_, (**e**) PHAN_1sphere_, (**f**) PHAN_20rod_.

The phantoms shown in Figure 1 were constructed in GATE Monte Carlo simulation by using the voxelized source and voxelized phantom to define the activity distribution and photon attenuation, respectively. PHAN_brain_ was the Hoffman 3D brain phantom. Figure 1a–c demonstrate the axial, coronal, and sagittal view of PHAN_brain_. To increase the dataset size and diversity for CNN training, PHAN_brain_ was slightly modified to generate 20 phantom configurations (2 translations× 2 rotations× 5 deformations), where each of them was filled with activity of 3.7 × 10^6^ Bq. PHAN_5rod_ was a cylinder of dimeter 50 mm, length of 80 mm and containing 5 rods with diameters of 2, 4, 6, 8, 10 mm. Figure 1d demonstrates the axial view of PHAN_5rod_. The target-to-background ratio (TBR) was set at 0, 2, 4, 5, 8, 10, 16, 20 to generate 8 phantom configurations, where the rod inserts within PHAN_5rod_ were filled with activity concentration of 1.69 × 10^6^ Bq/mL. PHAN_1sphere_ was a cylinder of diameter 50 mm, length of 80 mm and containing a 10-mm-diameter sphere. Figure 1e demonstrates the axial view of PHAN_1sphere_. The sphere within PHAN_1sphere_ was filled with water (i.e., cold sphere), while the cylinder was filled with activity concentration of 1.69 × 10^6^ Bq/mL. PHAN_20rod_ was an elliptical cylinder of major axis 55 mm, minor axis of 50 mm, length of 80 mm, and containing 20 rods with 2, 3, 4, 5 mm diameter. Figure 1f demonstrates the axial view of PHAN_20rod_. The white rod inserts within PHAN_20rod_ were filled with activity concentration of 1.69 × 10^6^ Bq/mL, while the gray rod inserts were filled with activity concentration of 8.44 × 10^5^ Bq/mL. Overall, a total of 30 phantom configurations were modeled in GATE Monte Carlo simulation, where each phantom configuration was simulated twice with a 20 min emission scan duration, once for Ga-68 (CNN input images) and once for back-to-back 511-keV gamma rays (CNN output images).

### 2.4. CNN Models for Positron Range Correction

**Figure 2:**
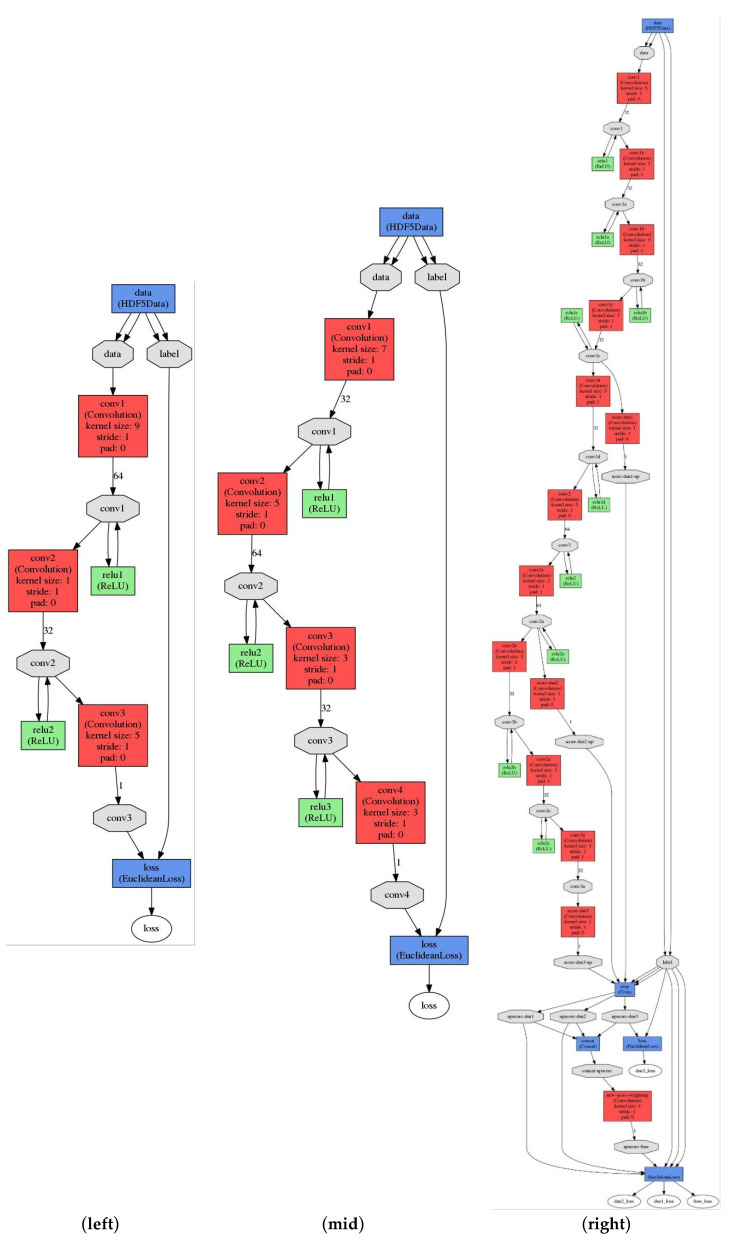
The architectures of CNN1 (**left**), CNN2 (**mid**) and CNN3 (**right**).

Figure 2 shows the architectures of CNN models used in this study to compensate positron range effects of Ga-68 in preclinical PET imaging. CNN1 was a 3-layered model proposed by Dong et al. for super-resolution recovery [15]. CNN2 was a 4-layered model proposed by Nie et al. for pseudo CT synthesis from MRI [16]. CNN3 was the deeply supervised nets (DSN) version of CNN2 to supervise features at each convolutional stage, enabled by layer-wise dense connections in both backbone networks and prediction layers [17]. Because the error distribution was expected to be Gaussian, the root mean square error (RMSE), i.e., the Euclidean distance, was used as the loss function to minimize the difference between Ga-68 PET images and the corresponding gamma source images. Using RMSE as the loss function favors a high peak signal-to-noise ratio (PSNR). The input images were prepared as 32 × 32-pixel sub-images randomly cropped from the original image. To avoid border effects, all the convolutional layers have no padding, and the network produces an output image with 20 × 20 matrix size for CNN1 and 18 × 18 matrix size for CNN2 and CNN3. The training datasets were sub-images extracted from the PET images of 16 PHAN_brain_ and 4 PHAN_5rod_ (TBR = 0, 4, 5, 8) with a stride of 14. The testing datasets were sub-images extracted from the PET images of 4 PHAN_brain_ (other than those used in CNN training) and 4 PHAN_5rod_ (TBR = 2, 10, 16, 20) with a stride of 21. The training and testing datasets provide roughly 111,078 and 25,774 sub-images, respectively. The filter weights of each layer were initialized by using Xavier initialization, which could automatically determine the scale of initialization based on the number of input and output neurons [18]. All biases were initialized with zero. The models were trained using stochastic gradient descent with mini-batch size of 128, learning rate of 0.01 and momentum of 0.9. The CNN models were built, trained and tested by using Caffe (Convolutional Architecture for Fast Feature Embedding) CNN platform (version 1.0.0-rc5 with CUDA 8.0.61) on an Ubuntu server (version 16.04.4 LTS) with two RTX 2080 (NVIDIA, Santa Clara, CA, USA) graphics cards [19].

### 2.5. Quantitative Analysis

The difference between Ga-68 PET images corrected by CNN-based positron range correction (I_PRC_) and the corresponding gamma source images (I_gamma_) was quantified by calculating the RMSE and PSNR:

(1) $$\mathrm{RMSE}=\sqrt{\frac{\sum_{i=1}^{V}{\left(I_{\mathrm{gamma}}-I_{\mathrm{PRC}}\right)}^{2}}{V}}$$

where V is the number of voxels within the whole image,

(2) $$\mathrm{PSNR}=20\log_{10}\frac{I_{\max}}{\mathrm{RMSE}}$$

where I_max_ is the maximum intensity value of the image. RMSE and PSNR provide a measure of image quality over the whole image.

The ability of Ga-68 PET images (I_Ga68_), I_gamma_, and I_PRC_ to recover contrast in small targets was quantified by calculating the recovery coefficient (RC), which was defined as:

(3) $$\mathrm{RC}=\frac{{\mathrm{AVG}}_{\mathrm{target}}}{{\mathrm{AVG}}_{\mathrm{uniform}}}$$

where AVG_target_ is the average of a small target, and AVG_uniform_ is the average of a uniform region. The coefficient of variation of RC (CV_RC_) was defined as:

(4) $${\mathrm{CV}}_{\mathrm{RC}}=\sqrt{{\left(\frac{{\mathrm{SD}}_{\mathrm{target}}}{{\mathrm{AVG}}_{\mathrm{target}}}\right)}^{2}+{\left(\frac{{\mathrm{SD}}_{\mathrm{uniform}}}{{\mathrm{AVG}}_{\mathrm{uniform}}}\right)}^{2}}$$

where SD_target_ is the standard deviation of a small target, and SD_uniform_ is the standard deviation of a uniform region. To calculate AVG_target_ and SD_target_, the image slices of PHAN_5rod_ over the central 50 mm length were averaged to obtain one average image, which was used to determine the voxel coordinate with maximum intensity for each rod. The pixel coordinates were then used to create a line profile along the axial direction. The AVG_target_ and SD_target_ were the average and standard deviation of pixel values in the line profile. As for AVG_uniform_ and SD_uniform,_ the image slices of PHAN_1sphere_ over the central 50 mm length were averaged to obtain one average image. A circular region-of-interest (ROI) with 10 mm diameter was placed on the cylinder of the average image to calculate AVG_uniform_ and SD_uniform_, corresponding to the average and standard deviation within the circular ROI, respectively.

The spill-over of activity in I_Ga68_, I_gamma_ and I_PRC_ was quantified by calculating the spill-over ratio (SOR), which was defined as:

(5) $$\mathrm{SOR}=\frac{{\mathrm{AVG}}_{\mathrm{cold}}}{{\mathrm{AVG}}_{\mathrm{hot}}}$$

where AVG_cold_ is the average of a cold spot, and AVG_hot_ is the average of a hot spot. The coefficient of variation of SOR (CV_SOR_) was defined as:

(6) $${\mathrm{CV}}_{\mathrm{SOR}}=\sqrt{{\left(\frac{{\mathrm{SD}}_{\mathrm{cold}}}{{\mathrm{AVG}}_{\mathrm{cold}}}\right)}^{2}+{\left(\frac{{\mathrm{SD}}_{\mathrm{hot}}}{{\mathrm{AVG}}_{\mathrm{hot}}}\right)}^{2}}$$

where SD_cold_ is the standard deviation of a cold spot, and SD_hot_ is the standard deviation of a hot spot. A 10-mm-diameter ROI was placed on the cold sphere of PHAN_1sphere_ in the slice of the sphere center to calculate AVG_cold_ and SDc_old_, corresponding to the average and standard deviation within the cold ROI, respectively. For the same image slice, a 10-mm-diameter ROI was placed on the cylinder of PHAN_1sphere_ to calculate AVG_hot_ and SD_hot_, corresponding to the average and standard deviation within the hot ROI, respectively.

## 3. Results

**Figure 3:**
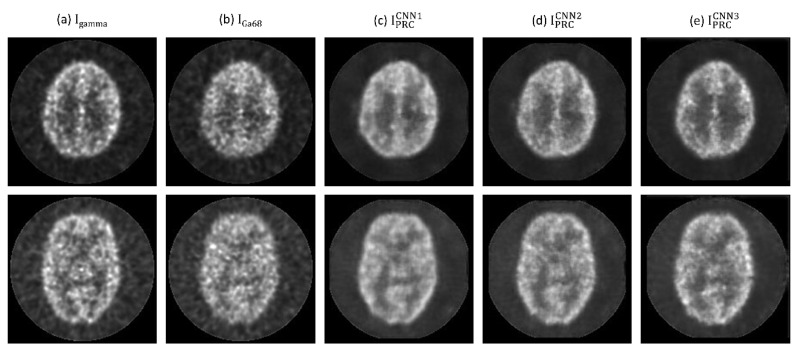
(**a**) I_gamma_, (**b**) I_Ga68_, (**c**) $I_{\mathrm{PRC}}^{\mathrm{CNN}1}$, (**d**) $I_{\mathrm{PRC}}^{\mathrm{CNN}2}$, (**e**) $I_{\mathrm{PRC}}^{\mathrm{CNN}3}$ of PHAN_brain_ at 2 different axial slices (**top row** and **bottom row**).

**Figure 4:**
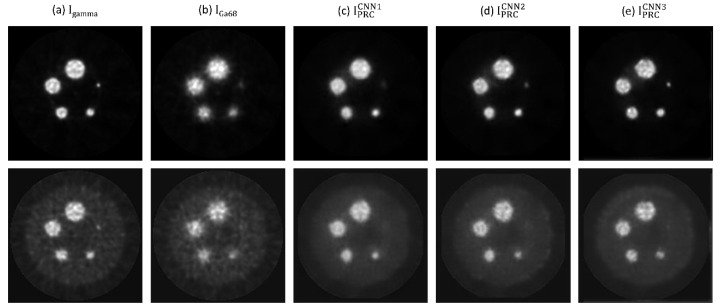
(**a**) I_gamma_, (**b**) I_Ga68_, (**c**) $I_{\mathrm{PRC}}^{\mathrm{CNN}1}$, (**d**) $I_{\mathrm{PRC}}^{\mathrm{CNN}2}$, (**e**) $I_{\mathrm{PRC}}^{\mathrm{CNN}3}$ of PHAN_5rod_ with TBR = 0 (**top row**) and TBR = 10 (**bottom row**).

**Figure 5:**
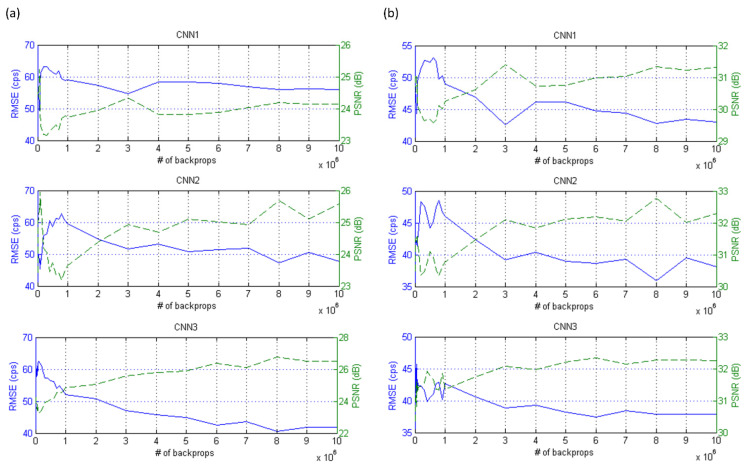
RMSE (solid line, left axis) and PSNR (dashed line, right axis) between I_gamma_ and I_PRC_ for (**a**) PHAN_brain_ and (**b**) PHAN_5rod_ with TBR = 10 (**top row**: CNN1-based correction; **mid row**: CNN2-based correction; **bottom row**: CNN3-based correction).

Figure 3 demonstrates I_gamma_, I_Ga68_, and Ga-68 PET images after positron range correction based on CNN1 ($I_{\mathrm{PRC}}^{\mathrm{CNN}1}$), CNN2 ($I_{\mathrm{PRC}}^{\mathrm{CNN}2}$) and CNN3 ($I_{\mathrm{PRC}}^{\mathrm{CNN}3}$) of PHAN_brain_. With naked eye observation, boundaries in Ga-68 PET images became sharper after correction. Figure 4 demonstrates I_gamma_, I_Ga68_, $I_{\mathrm{PRC}}^{\mathrm{CNN}1}$, $I_{\mathrm{PRC}}^{\mathrm{CNN}2}$, $I_{\mathrm{PRC}}^{\mathrm{CNN}3}$ of PHAN_5rod_ with TBR = 0 and 10. The 2-mm rod in PHAN_5rod_ with TBR = 0 that was barely seen in I_Ga68_ became visible in $I_{\mathrm{PRC}}^{\mathrm{CNN}2}$ and $I_{\mathrm{PRC}}^{\mathrm{CNN}3}$. The RMSE and PSNR between I_gamma_ and I_PRC_ are shown in Figure 5a for PHAN_brain_ and Figure 5b for PHAN_5rod_ with TBR = 10. Lower RMSEs and higher PSNRs were observed in Figure 5b than those in Figure 5a. For either phantom configuration, the lowest RMSE and the highest PSNR were found in CNN3-based correction, followed by CNN2- and CNN1-based correction.

**Figure 6:**
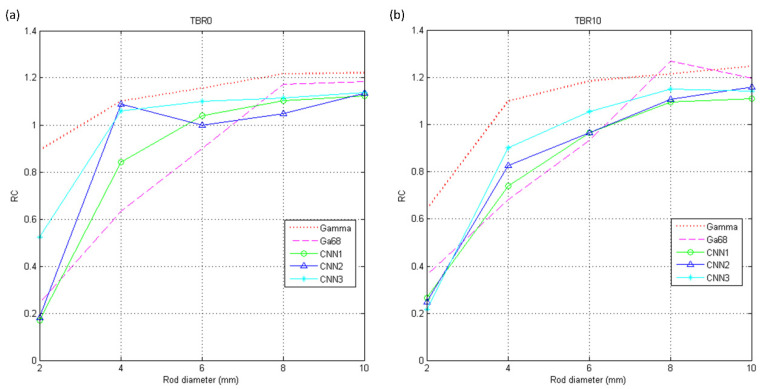
RC of I_gamma_, I_Ga68_, $I_{\mathrm{PRC}}^{\mathrm{CNN}1}$, $I_{\mathrm{PRC}}^{\mathrm{CNN}2}$, $I_{\mathrm{PRC}}^{\mathrm{CNN}3}$ using PHAN_5rod_ with (**a**) TBR = 0 and (**b**) TBR = 10.

**Figure 7:**
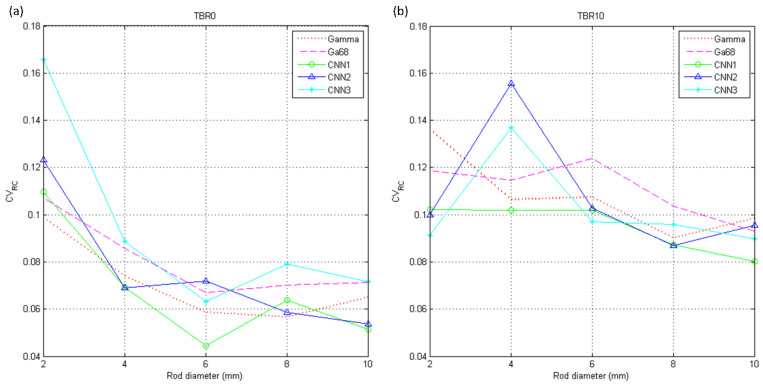
CV_RC_ of I_gamma_, I_Ga68_, $I_{\mathrm{PRC}}^{\mathrm{CNN}1}$, $I_{\mathrm{PRC}}^{\mathrm{CNN}2}$, $I_{\mathrm{PRC}}^{\mathrm{CNN}3}$ using PHAN_5rod_ with (**a**) TBR = 0 and (**b**) TBR = 10.

The RCs of I_gamma_, I_Ga68_, $I_{\mathrm{PRC}}^{\mathrm{CNN}1}$, $I_{\mathrm{PRC}}^{\mathrm{CNN}2}$, $I_{\mathrm{PRC}}^{\mathrm{CNN}3}$ are shown in Figure 6a for PHAN_5rod_ with TBR = 0 and Figure 6b for PHAN_5rod_ with TBR = 10. The RCs of positron range corrected images were higher in TBR = 0 than those in TBR = 10 for 4- and 6-mm rods, while the difference was less obvious in 8- and 10-mm rods. Among three corrected images, the highest RCs were observed in $I_{\mathrm{PRC}}^{\mathrm{CNN}3}$ for 6-, 8-, and 10-mm rods in TBR = 0, while the highest RC for 4-mm rod was observed in $I_{\mathrm{PRC}}^{\mathrm{CNN}2}$. With regard to TBR = 10, the highest RCs were observed in $I_{\mathrm{PRC}}^{\mathrm{CNN}3}$ for 4-, 6-, and 8-mm rods, while the highest RC for 10-mm rod was observed in $I_{\mathrm{PRC}}^{\mathrm{CNN}2}$. The CV_RC_s of I_gamma_, I_Ga68_, $I_{\mathrm{PRC}}^{\mathrm{CNN}1}$, $I_{\mathrm{PRC}}^{\mathrm{CNN}2}$, $I_{\mathrm{PRC}}^{\mathrm{CNN}3}$ were shown in Figure 7a for PHAN_5rod_ with TBR = 0 and Figure 7b for PHAN_5rod_ with TBR = 10. The CV_RC_s of positron range corrected images were lower in TBR = 0 than those in TBR = 10 for 4-, 6-, 8-, and 10-mm rods. Among three corrected images, the CV_RC_s of $I_{\mathrm{PRC}}^{\mathrm{CNN}1}$ were slight lower than those from $I_{\mathrm{PRC}}^{\mathrm{CNN}2}$ and $I_{\mathrm{PRC}}^{\mathrm{CNN}3}$ for either phantom configuration.

**Figure 8:**
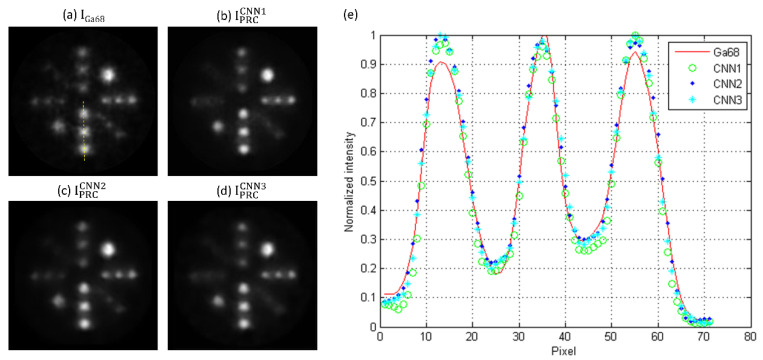
(**a**) I_Ga68_, (**b**) $I_{\mathrm{PRC}}^{\mathrm{CNN}1}$, (**c**) $I_{\mathrm{PRC}}^{\mathrm{CNN}2}$, (**d**) $I_{\mathrm{PRC}}^{\mathrm{CNN}3}$ of PHAN_20rod_ and (**e**) comparison of intensity profiles through the dashed line in (**a**).

With regard to the spillover effect determined by using PHAN_1sphere_, the SORs of I_gamma_, I_Ga68_, $I_{\mathrm{PRC}}^{\mathrm{CNN}1}$, $I_{\mathrm{PRC}}^{\mathrm{CNN}2}$, $I_{\mathrm{PRC}}^{\mathrm{CNN}3}$ were 0.017, 0.026, 0.021, 0.020, and 0.020, respectively, while the corresponding CV_SOR_s were 0.454, 0.424, 0.406, 0.416, and 0.441. Figure 8 demonstrates I_Ga68_, $I_{\mathrm{PRC}}^{\mathrm{CNN}1}$, $I_{\mathrm{PRC}}^{\mathrm{CNN}2}$, $I_{\mathrm{PRC}}^{\mathrm{CNN}3}$ of PHAN_20rod_ and comparison of intensity profiles through the dashed line. Sharper boundaries were observed in 4- and 5-mm rods after positron range correction. On the other hand, the image quality improvement was limited in 2- and 3-mm rods, except for the 3-mm rod with 8.44 × 10^5^ Bq/mL in $I_{\mathrm{PRC}}^{\mathrm{CNN}2}$ and $I_{\mathrm{PRC}}^{\mathrm{CNN}3}$.

## 4. Discussion

The quantitative capabilities of PET are confounded by a number of degrading factors, whereas the most prominent factors are low signal-to-noise ratio and intrinsically limited spatial resolution [20]. Tumor-targeted theranostic approaches have high lesion-to-background ratio. For example, PET imaging with Ga-68 labeled somatostatin analogues shows high affinity for tumors expressing somatostatin receptors [7,8]. However, the image blurring caused by the positron range effect of Ga-68 may impact the accuracy of treatment planning based on Ga-68 PET imaging. Fourier devolution techniques have been applied to compensate the positron range effects in PET imaging [21], which inspired us to investigate the possibility of using CNN methods for positron range correction. According to Herraiz et al. [22], their study published in 2021 was the first work to successfully combine deep learning and positron range correction in a coherent framework. In our opinion, more studies are needed in this field. Hence, we investigated the feasibility of positron range correction based on three different CNN models in preclinical PET imaging of Ga-68.

Song et al. have presented a work to recover high-resolution PET image from its low-resolution version by using CNN-based approaches for F-18 FDG exams [23]. A 3-layer CNN model proposed by Dong et al. [15], i.e., CNN1, and a 20-layer CNN model proposed by Kim et al. [24] were adapted in their work. The low-resolution images used as the CNN inputs were acquired with Siemens HR+ scanner, while the high-resolution images used as the CNN labels were acquired with Siemens HRRT scanner, a high-resolution dedicated brain PET scanner. Two simulation studies using the BrainWeb digital phantom and a clinical patient study were conducted. They concluded that adding additional channels that extract anatomical features from MRI could improve the performance of CNN-based resolution recovery methods, whereas deep CNNs outperform shallow CNNs. Since the positron range effect would result in image blurring, it is intuitively reasonable to expect that CNN models designed for resolution recovery may be potential candidate for positron range correction in Ga-68 PET imaging. Hence, CNN1 was adapted in our study.

Herraiz et al. have presented a work which adapts the U-Net network to correct positron range effects of Ga-68 in preclinical PET imaging [22]. In their work, the input data to CNN were Ga-68 images, while the label data were the F-18 images. The PET images for CNN training and testing were generated by using the Monte Carlo simulator MCGPU-PET to model data acquisition in an Inveon PET/CT scanner. Their results demonstrated that their proposed method was able to restore the PET images going from 60% up to 95% while maintain low noise levels. They concluded that it is sufficient to use PET images without the corresponding CT as input for the neural network, and including not only the reference slice but also some additional neighbor slices could improve the CNN-based positron range correction method. In our opinion, Herraiz et al. demonstrated that CNN models suitable for positron range correction were not only limited to those designed for resolution recovery, because the U-Net network was originally designed for image segmentation [25]. Positron range correction is inherently an ill-posed problem, because there are multiple Ga-68 activity distributions that may correspond to the same blurred image. Pseudo CT synthesis from MRI is also proposed to solve ill-posed problem, because there are multiple MRI values that may correspond to the same CT value. It was hypothesized that CNN models designed for pseudo CT synthesis from MRI may be potential candidate for positron range correction, so CNN2 and CNN3 were adapted in our study.

In Reference [22], it was assumed that the reconstruction method already incorporated positron range correction for F-18, and their image data for CNN training, testing, and validation were generated from numerical models of mice from a repository. In this work, the CNN output images were back-to-back 511-keV gamma rays, which were not affected by the positron range effects. Hence, our method can be used in PET scanners without F-18 positron range correction. NEMA performance measurements have been well accepted by the manufacturers, and most major companies now specify their product performance in terms of these standardized and traceable specifications. This approach to performance documentation facilitates quantitative comparison of cameras by the user with the assurance that all reported values are measured in the same way and, therefore, are directly comparable [26,27]. Hence, a modified NEMA protocol was used in this study to evaluate the performance of CNN-based positron range correction in terms of resolution recovery and spill-over. Our results demonstrated that the image quality of Ga-68 images was improved after positron range correction based on the 3 CNN models investigated in this work, while CNN3 outperformed CNN1 and CNN2 qualitatively and quantitatively. With regard to qualitative observation, it was found that boundaries in Ga-68 images became sharper after correction (see Figure 3, Figure 4 and Figure 8). As for quantitative analysis, the RC and SOR were increased after correction, while no substantial increase in CV_RC_ or CV_SOR_ was observed. Overall, CNN3 should be a good candidate architecture for positron range correction in Ga-68 preclinical PET imaging.

Several limitations to this study need to be acknowledged. First, the data acquisition, processing and reconstruction approaches can influence the study results. The protocol parameters used in this study were suggested by the manufacturers and are currently employed in a real scanner installed in our institution. Second, all images were generated from Monte Carlo simulations. Since it is difficult to obtain PET images without positron range effect from real experiments, Monte Carlo simulation was used to generate Ga-68 images and corresponding gamma source images for CNN training and testing. Third, the impact of image blurring caused by positron range effect on the accuracy of treatment planning based on Ga-68 was not investigated. Assessments of the proposed methods in real Ga-68 images and the resulting impact on treatment planning for Lu-177 radionuclide therapy need to be further investigated.

## 5. Conclusions

This study investigated the feasibility of positron range correction based on three different CNN models in preclinical PET imaging of Ga-68. CNN1 was a model originally designed for super-resolution recovery, while CNN2 and CNN3 were models originally designed for pseudo CT synthesis from MRI. Monte Carlo simulation was used to generate Ga-68 images and corresponding gamma source images for CNN training and testing. According to our results, CNN3 outperformed CNN1 and CNN2 qualitatively and quantitatively. With regard to qualitative observation, it was found that boundaries in Ga-68 images became sharper after correction. As for quantitative analysis, the RC and SOR were increased after correction, while no substantial increase in CV_RC_ or CV_SOR_ was observed. Overall, CNN3 should be a good candidate architecture for positron range correction in Ga-68 preclinical PET imaging.

## References

[1] Herfert K., Mannheim J.G., Kuebler L., Marciano S., Amend M., Parl C., Napieczynska H., Maier F.M., Vega S.C., Pichler B.J. (2020). Quantitative Rodent Brain Receptor Imaging. Mol. Imaging Biol..

[2] Wierstra P., Sandker G., Aarntzen E., Gotthardt M., Adema G., Bussink J., Raavé R., Heskamp S. (2019). Tracers for non-invasive radionuclide imaging of immune checkpoint expression in cancer. EJNMMI Radiopharm. Chem..

[3] Walter M.A., Hildebrandt I.J., Hacke K., Kesner A.L., Kelly O., Lawson G.W., Phelps M.E., Czernin J., Weber W.A., Schiestl R.H. (2010). Small-animal PET/CT for monitoring the development and response to chemotherapy of thymic lymphoma in Trp53−/− mice. J. Nucl. Med. Off. Publ. Soc. Nucl. Med..

[4] Moses W.W. (2011). Fundamental Limits of Spatial Resolution in PET. Nucl. Instrum. Methods Phys. Res. Sect. A.

[5] Kuntner C., Stout D. (2014). Quantitative preclinical PET imaging: Opportunities and challenges. Front. Phys..

[6] Payolla F., Massabni A., Orvig C. (2019). Radiopharmaceuticals for diagnosis in nuclear medicine: A short review. Eclética Química J..

[7] Baum R.P., Kulkarni H.R. (2012). THERANOSTICS: From Molecular Imaging Using Ga-68 Labeled Tracers and PET/CT to Personalized Radionuclide Therapy—The Bad Berka Experience. Theranostics.

[8] Emmett L., Willowson K., Violet J., Shin J., Blanksby A., Lee J. (2017). Lutetium (177) PSMA radionuclide therapy for men with prostate cancer: A review of the current literature and discussion of practical aspects of therapy. J. Med. Radiat. Sci..

[9] St. James S., Bednarz B., Benedict S., Buchsbaum J.C., Dewaraja Y., Frey E., Hobbs R., Grudzinski J., Roncali E., Sgouros G. (2021). Current Status of Radiopharmaceutical Therapy. Int. J. Radiat. Oncol. Biol. Phys..

[10] Malcolm J., Falzone N., Lee B.Q., Vallis K.A. (2019). Targeted Radionuclide Therapy: New Advances for Improvement of Patient Management and Response. Cancers.

[11] Lassmann M., Eberlein U. (2018). The Relevance of Dosimetry in Precision Medicine. J. Nucl. Med. Off. Publ. Soc. Nucl. Med..

[12] Conti M., Eriksson L. (2016). Physics of pure and non-pure positron emitters for PET: A review and a discussion. EJNMMI Phys..

[13] Arabi H., AkhavanAllaf A., Sanaat A., Shiri I., Zaidi H. (2021). The promise of artificial intelligence and deep learning in PET and SPECT imaging. Phys. Med..

[14] Jan S., Santin G., Strul D., Staelens S., Assié K., Autret D., Avner S., Barbier R., Bardiès M., Bloomfield P.M. (2004). GATE: A simulation toolkit for PET and SPECT. Phys. Med. Biol..

[15] Dong C., Loy C.C., He K., Tang X. (2016). Image Super-Resolution Using Deep Convolutional Networks. IEEE Trans. Pattern Anal. Mach. Intell..

[16] Nie D., Cao X., Gao Y., Wang L., Shen D. Estimating CT Image from MRI Data Using 3D Fully Convolutional Networks. Proceedings of the Deep Learning and Data Labeling for Medical Applications: First International Workshop, LABELS 2016, and Second International Workshop, DLMIA 2016, Held in Conjunction with MICCAI 2016.

[17] Lee C.-Y., Xie S., Gallagher P., Zhang Z., Tu Z. (2014). Deeply-Supervised Nets. arXiv.

[18] Glorot X., Bengio Y. Understanding the difficulty of training deep feedforward neural networks. Proceedings of the Thirteenth International Conference on Artificial Intelligence and Statistics.

[19] Jia Y., Shelhamer E., Donahue J., Karayev S., Long J., Girshick R., Guadarrama S., Darrell T. Caffe: Convolutional Architecture for Fast Feature Embedding. Proceedings of the 2014 ACM Conference on Multimedia.

[20] Rogasch J.M.M., Hofheinz F., Lougovski A., Furth C., Ruf J., Großer O.S., Mohnike K., Hass P., Walke M., Amthauer H. (2014). The influence of different signal-to-background ratios on spatial resolution and F18-FDG-PET quantification using point spread function and time-of-flight reconstruction. EJNMMI Phys..

[21] Derenzo S.E. (1986). Mathematical Removal of Positron Range Blurring in High Resolution Tomography. IEEE Trans. Nucl. Sci..

[22] Herraiz J.L., Bembibre A., López-Montes A. (2021). Deep-Learning Based Positron Range Correction of PET Images. Appl. Sci..

[23] Song T.A., Chowdhury S.R., Yang F., Dutta J. (2020). Super-Resolution PET Imaging Using Convolutional Neural Networks. IEEE Trans. Comput. Imaging.

[24] Kim J., Lee J.K., Lee K.M. Accurate Image Super-Resolution Using Very Deep Convolutional Networks. Proceedings of the 2016 IEEE Conference on Computer Vision and Pattern Recognition (CVPR).

[25] Ronneberger O., Fischer P., Brox T. (2015). U-Net: Convolutional Networks for Biomedical Image Segmentation. arXiv.

[26] Raff U., Spitzer V.M., Hendee W.R. (1984). Practicality of NEMA performance specification measurements for user-based acceptance testing and routine quality assurance. J. Nucl. Med. Off. Publ. Soc. Nucl. Med..

[27] Teuho J., Riehakainen L., Honkaniemi A., Moisio O., Han C., Tirri M., Liu S., Grönroos T.J., Liu J., Wan L. (2020). Evaluation of image quality with four positron emitters and three preclinical PET/CT systems. EJNMMI Res..

